# Stereotactic Bony Trajectory Preservation for Responsive Neurostimulator Lead Placement Following Depth EEG Recording

**DOI:** 10.7759/cureus.549

**Published:** 2016-03-30

**Authors:** Kai Miller, Casey H Halpern

**Affiliations:** 1 Department of Neurosurgery, Stanford University School of Medicine

**Keywords:** depth electroencephalography, responsive neurostimulation, epilepsy

## Abstract

Responsive neurostimulation (RNS) is rapidly gaining traction as a therapy for medically refractory epilepsy. Depth electrode placement for stimulation of a deep seizure focus may be indicated after the focus has been electrophysiologically localized using depth electroencephalography (depth EEG). We describe a simple technique whereby the bony trajectories created during initial stereotactic placement of depth EEG electrodes are preserved and reused for RNS with depth electrodes. This technique may help to improve targeting and maximize surgical efficiency.

## Introduction

**Figure 1:**
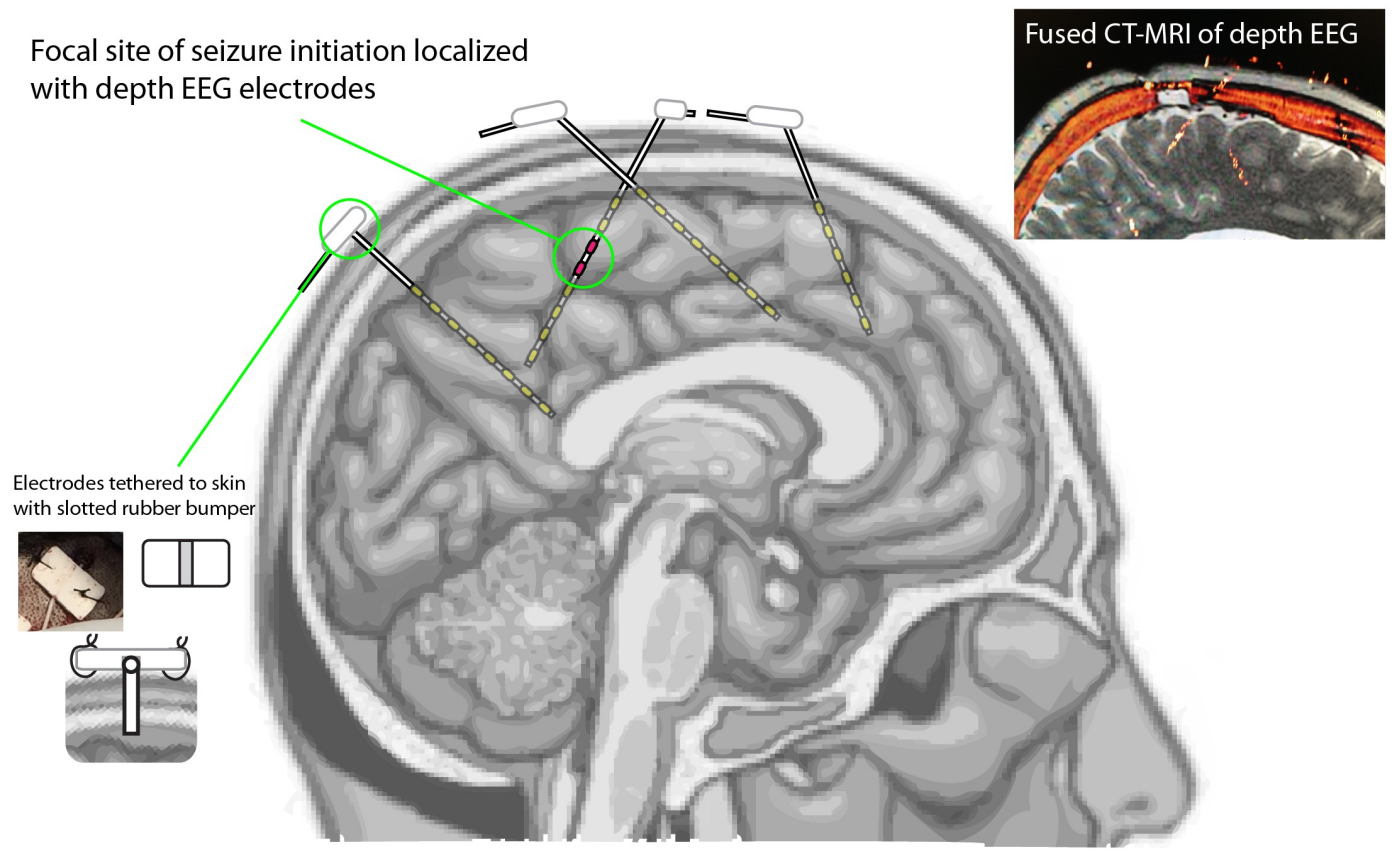
Depth EEG Intracortical Recording Identifies Seizure Onset Locus

Treatment for epilepsy is complex and evolving. Classically, it begins with medical management, first with a single antiepileptic drug (AED), then another, escalating to multi-AED therapy [1]. Those who do not respond to medications may undergo laser-ablation or surgical resection of the epileptic focus, or receive a vagal nerve stimulator (VNS) as an adjuvant therapy [1-2]. Localization of the seizure focus prior to ablation or resection is often attempted using implanted depth EEG electrodes (Figure 1) [3-4]. If the identified seizure focus lies within a region of motor or speech eloquence, lesioning or resection may not be possible [5]. Likewise, patients with epileptic foci localized to the mesial temporal lobe are occasionally excluded from lesioning or resection in the case of bilateral disease, verbal decline during selective amytal injection (WADA test), or global memory deficit during a WADA test [6-7]. For these patients, a new therapeutic intervention has emerged: responsive electrical stimulation of the seizure focus [8-12]. Following depth EEG monitoring, it is most often desirable to implant stimulating electrodes at the precise site through the identical bony entrance and bony trajectory where a depth EEG recording electrode showed seizure initiation. We present a simple technique for preserving depth EEG trajectory for responsive neurostimulator lead placement. Informed consent was obtained from the patient for this procedure.

## Technical report

We describe a simple technique whereby the stereotactic depth EEG trajectory through skull and brain tissue is reused for intracortical RNS.

### Depth EEG lead placement

**Figure 2:**
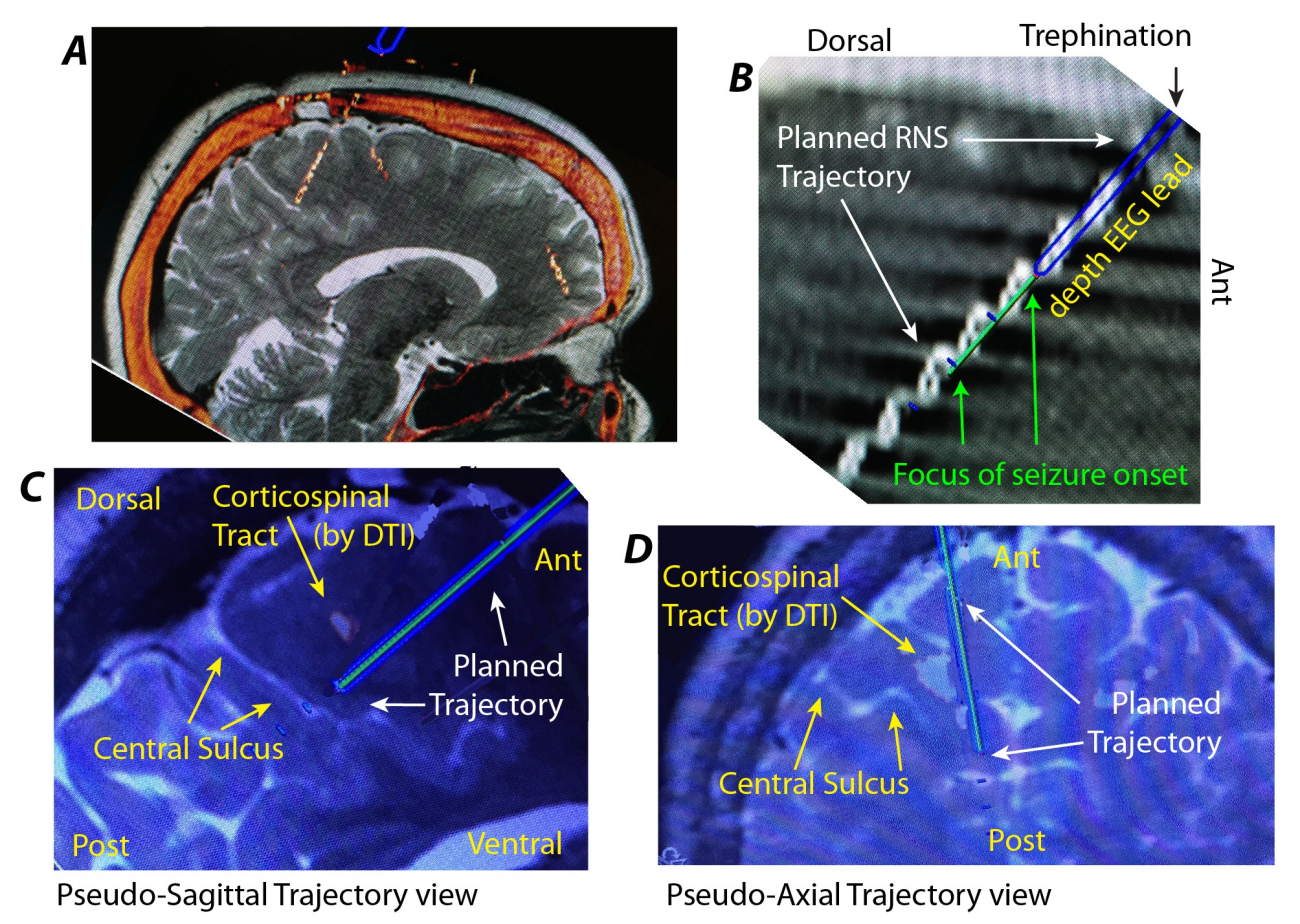
Stereotactic Planning *(A) *Sagittal slice through fused CT & MRI, showing depth EEG electrodes in situ. *(B)* The trajectory for the RNS electrodes is determined by planning a trajectory that passes directly through the trephination and terminates at the depth EEG site of seizure focus identification. *(C)* Anatomical MRIs as well as specialized scans such at diffusion tractography are fused to the base-CT, and allow direct comparison with planned trajectory. A pseudo-sagittal trajectory view is shown.* (D) *As in (C), but for a pseudo-axial trajectory view.

Bone fiducials are used for improved registration during stereotactic guidance of depth EEG electrodes. They are placed on the day or morning prior to surgery, and a computed tomography (CT) is obtained (used as the base/reference - “base-CT”). Anatomical magnetic resonance images (MRIs), positron emission tomography (PET) scans, magnetoencephalography (MEG), and EEG are reviewed in a meeting with the epilepsy neurologist on the day prior to depth EEG placement, and trajectories are planned using the Framelink application within the Stealth Navigation system (Medtronic, Minneapolis, MN, USA, illustrated in Figure 2).

The Vertek Articulating Arm (Medtronic) is used in concert with the Precision Aiming Device (PAD) (Medtronic) and Vertek probe (VP) (Medtronic) to align depth EEG trajectories to stereotactic plan, in a manner similar to practice for common biopsy using the Medtronic Suite (Medtronic, Minneapolis, MN, USA). Small caliber holes are obtained by passing the drill through the PAD with the aid of a reducer, so that the trephination is aligned with the desired electrode trajectory [13]. After verifying alignment with the VP, a rigid, slotted, cannula is passed through the PAD (using a different reducer), and is therefore dually fixated by the PAD and the bony hole. Depth EEG electrode leads are passed through the slotted cannula to pre-determined depth, and held rigidly at the level of the skin for the percutaneuous placement while the cannula is removed. Electrode leads are anchored to a rubber bumper, and rigidly tethered to the skin, first with staples, then with suture (illustrated in bottom left inset of Figure 1).

### RNS intracortical lead placement

**Figure 3:**
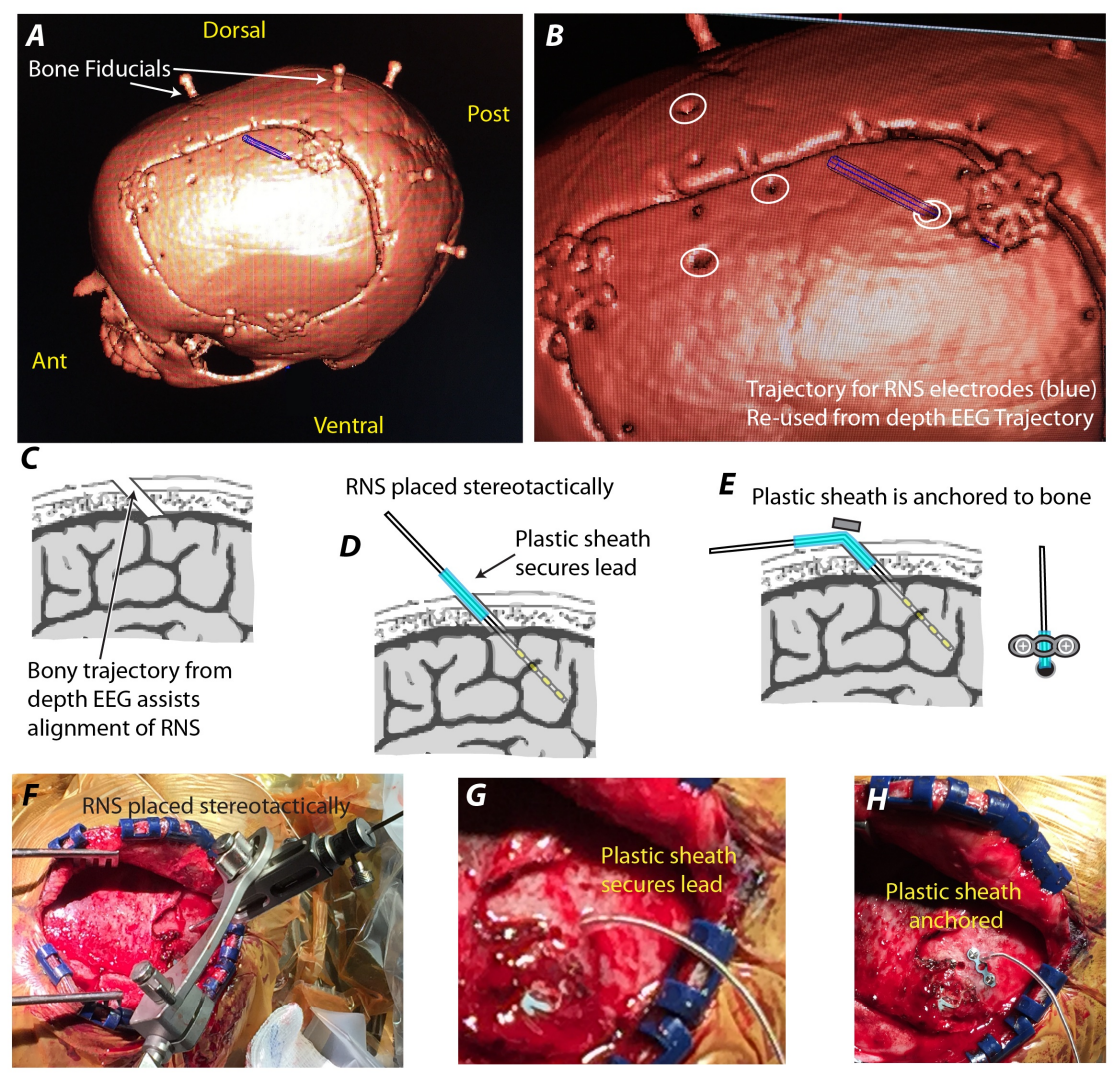
Technique for RNS Intracortical Lead Placement *(A) *Bone fiducials are used for stereotactic guidance of both depth EEG as well as RNS lead placement. (Note that craniotomy is from prior grid placement in this patient and unrelated in present context.) Trajectory of depth EEG for lead that defined seizure onset locus is shown in blue. *(B) *As in (A), trajectory of depth EEG for lead that defined seizure onset locus is shown in blue. White circles show bone trephinations for other depth EEG leads. *(C) *The bony path of the prior lead placement (trephination) assists with RNS lead placement. Because of the thin cross-section (2.1 mm) compared with the skull thickness, the depth EEG trajectory is reproduced in a brute force fashion. *(D)* A plastic sheath anchors the RNS lead within the trephination. *(E)* The plastic sheath is anchored to the bone.* (F) *Intraoperative photograph of RNS lead placement using the Precision Aiming Device with the Vertek Arm under stereotactic guidance. *(G) *The plastic sheath anchors the RNS lead within the trephination. *(H)* The plastic sheath is anchored to the bone using a 3-hole plate.

Bone fiducials are used for stereotactic guidance of RNS lead placement as they are for the phase 2 depth electrodes. They are placed on the day prior to surgery, and a CT is obtained (used as the base/reference - “base-CT”). Anatomical MRIs as well as specialized scans such at diffusion tractography are fused to the base-CT using the Framelink application within the Stealth Navigation system. The previous postoperative CT from the monitoring period, showing depth EEG electrodes in situ (“EEG-CT”) is also fused to the base-CT. The trajectory for the RNS electrodes is determined by planning a trajectory that passes directly through the trephination and terminates at the depth EEG site of seizure focus identification (Figure 3C). Note that reusing the depth EEG preoperative plan will be less accurate, in many cases, due to intraoperative variation from planned trajectories.

Intraoperatively for placement of the RNS lead, a skin incision is made that incorporates both the trephination trajectory as well as an exposed portion of the skull where the craniotomy will be made for placement of the RNS stimulator ferrule (NeuroPace, Mountain View, CA, USA). A cannula is then passed through the planned trajectory, using the PAD under stereotactic guidance. The existing trephination forces accurate reproduction of the depth EEG path. Because of the thin cross-section (2.1 mm) compared with the skull thickness (typically 10-13 mm), the depth EEG trajectory is reproduced in a physically direct, mechanically constrained fashion. The RNS depth electrode is then passed through the cannula to the target with a DL-330-3.5-K NeuroPace depth electrode (4 electrodes per lead, 3.5 mm spacing), confirming depth to target at the skull surface. A plastic sheath identical in diameter to the trephination is used to anchor the RNS lead within the trephination (Figures 3D, 3E). The plastic sheath is then anchored to the bone using a low profile 3- or 4-hole plate. The RNS lead is then inserted into the RNS stimulator, and the RNS stimulator is placed within a craniotomy in the standard fashion.

## Discussion

It is important that there be a seamless transition from diagnostic workup to therapeutic intervention in epilepsy. This requires close collaboration and interaction between neurologists and neurosurgeons at every stage. If neurologists determine in the course of their electrophysiological monitoring that a seizure focus has been directly captured by a depth electrode, then this technique is indicated. However, this technique would not be applicable if the focus is determined to be near, but not directly at, a depth EEG electrode site.

The technique we describe here helps to streamline the surgical intervention by creating two points of rigid fixation for the cannula though which the stimulating electrodes are placed, reducing and correcting minor errors in trajectory associated with the Vertek arm. The plastic sheath with 3-hole plate anchor maintains fixation without need for a burrhole-plug anchor and convex cover (with raised profile) at each insertion site that is used more commonly with responsive neurostimulation (e.g. with the NeuroPace device). Additionally, this technique circumvents the need for new holes in the skull.

## Conclusions

This technique represents a simple and low-profile technical variant that we feel will improve accuracy, minimize invasiveness, and streamline surgical intervention. Steps such as this preservation of bony trajectory will optimize efficiency as stereotactic surgery for epilepsy becomes more frequently performed. Furthermore, it will maximize the probability that stimulation for seizure abortion is delivered to the precise location where epileptic activity has been identified with depth EEG.
